# Non‐factor therapies for bleeding disorders: A primer for the general haematologist

**DOI:** 10.1002/jha2.442

**Published:** 2022-04-28

**Authors:** Dawn Swan, Johnny Mahlangu, Jecko Thachil

**Affiliations:** ^1^ National University Ireland Galway Republic of Ireland; ^2^ Department of Molecular Medicine and Haematology School of Pathology Faculty of Health Sciences University of the Witwatersrand and NHLS Johannesburg South Africa; ^3^ Department of Haematology Manchester University Hospitals NHS Foundation Trust Manchester UK

**Keywords:** concizumab, emicizumab, fitusiran, haemophilia, von Willebrand disease

## Abstract

Management of patients with severe bleeding disorders, particularly haemophilia A and B, and to a lesser extent, von Willebrand disease, has come on leaps and bounds over the past decade. Until recently, patients relied upon the administration of factor concentrates to prevent or treat bleeding episodes. Factor administration requires intravenous access and, in up to one‐third of patients, leads to the development of neutralising antibodies, or inhibitors, which are associated with more frequent bleeding episodes and higher morbidity. Novel non‐factor therapies may offer a solution to these unmet needs. In this review, we discuss the factor mimetics, particularly emicizumab, and the rebalancing agents, which inhibit antithrombin, tissue factor pathway inhibitor and activated protein C, and novel treatments to enhance von Willebrand factor levels. We review the available trial data, unanswered questions and challenges associated with these new treatment modalities. Finally, we provide practical management algorithms to aid the general haematologist when faced with a patient receiving emicizumab who requires surgery or may develop bleeding.

## 1 INTRODUCTION

Congenital haemophilia A (HA) and haemophilia B (HB) are X‐linked inherited bleeding disorders caused by mutations or deletions of the genes encoding clotting factor VIII (FVIII) and factor IX (FIX), respectively. Persons with haemophilia (PWH), particularly those with severe disease, defined by a plasma FVIII or FIX level less than 0.01 IU/ml (or <1%), are at risk of spontaneous life‐threatening bleeds and long‐term disability as a sequela of recurrent haemarthroses. Treatments for haemophilia started with cryoprecipitate in the middle part of the 20th century, followed by plasma‐derived factor FVIII or FIX concentrates. The subsequent introduction of purified, virus‐inactivated plasma products and recombinant factor replacement ushered in the era of prophylactic treatment, which significantly reduced bleeding rates and associated morbidity [1, 2, 3]. Extended half‐life products (EHL) have been engineered to utilise Fc‐fusion, albumin‐fusion or PEGylation to extend circulating biological half‐life and reduce the frequency of administration [4, 5, 6, 7, 8, 9].

von Willebrand disease (VWD) is an inherited bleeding disorder characterized by quantitative and or qualitative defects in von Willebrand factor (VWF), a large multimeric protein, which plays a critical role in primary haemostasis [10]. VWD is more common than haemophilia, with an estimated prevalence of up to 1% [11]. Recent therapeutic advances in the treatment of VWD have been slow compared with the evolution of haemophilia treatment, and patients with severe disease are still generally reliant upon plasma‐derived VWF containing concentrates or recombinant VWF where available, for treatment or prevention of bleeds [12, 13].

## 2 WHY DO WE NEED NON‐FACTOR THERAPIES?

Traditional factor replacement therapies have several disadvantages. To reduce the spontaneous bleeding risk to an acceptable level, a minimum FVIII or FIX level of 1%–3% has generally been targeted [14]. However, this does not prevent spontaneous bleeds in all patients. One study reported bleeds in some patients with trough levels >3% and conversely, no bleeds in others with levels <1%, suggesting the importance of additional unaccounted‐for haemostatic regulators [15]. A study of joint bleeds in mild–moderate HA patients concluded that a baseline FVIII level of 15% would prevent all bleeds [16], with another study by the same group suggesting a class of 12% to achieve this goal [17]. Provision of acceptable factor trough levels has traditionally been achieved via one of the three methods, wherein factor is administered one, two or three times per week [18]. Less‐frequent administration requires larger doses, producing higher peak levels and more extended periods with lower troughs. Given elevated FVIII is a known thrombosis risk, patients may swing between prothrombotic and pro‐haemorrhagic states [19]. Thrombosis is uncommon in patients with inherited bleeding disorders. More active patients require more frequent dosing or regimens tailored around training regimens or sporting fixtures. The use of pharmacokinetics can assist in dosing of factor replacement regimens, but avoidance of peaks and troughs altogether is not possible [20]. A sustained period of good haemostatic levels is also thought to prevent ‘micro‐bleeds’, which patients experience as joint niggles rather than bleeds and may impact their lifestyle. PWH may also need to adapt their activities around factor administration times (e.g., sports activities or long‐distance travel), which can have a significant psychosocial impact.

Frequency of administration often leads to problems securing venous access, with indwelling venous access devices commonly required, particularly in younger paediatric patients. Such devices require specialist training regarding the use and carry a risk of infection [21] and line‐related thrombosis, even in PWH [22, 23]. Venous access is particularly important in patients with inhibitors. Inhibitors are neutralising antibodies directed against exogenous factor replacement. They occur in approximately 25%–30% of severe HA and 3%–5% of HB and are predisposed by early and intensive factor exposure [24, 25, 26]. Current management of bleeds in inhibitor patients requires bypassing agents (recombinant activated factor VII [rFVIIa] or activated prothrombin complex concentrate [aPCC]). These agents confer a reported risk of 4%–6.5% of thrombosis and are less effective at securing haemostasis than standard factor replacement in the absence of inhibitors [27]. Immune tolerance induction (ITI) may successfully eradicate inhibitors in a proportion of patients but requires intensive, daily factor administration for months at a time [28]. As ITI is usually undertaken in childhood, treatment burden and associated hospital visits can adversely affect schooling and social development.

Further advances in therapeutic options for PWH and VWD are required to address these ongoing issues. In this review, we will discuss recent pharmacological developments, particularly focusing on factor mimetics, which perform the function of the deficient factor rather than simply replacing the factor itself, and rebalancing agents, which inhibit naturally occurring anticoagulants to promote haemostasis. Discussion of gene therapy is outside the scope of this review.

## 3 NON‐FACTOR THERAPY—MIMETICS

Mimetics of coagulation factors could provide a similar (or better) haemostatic potential to that achieved by factor replacement. The most well‐established factor mimetic is emicizumab. Emicizumab (Hemlibra, Roche) is a humanised bispecific IgG4 monoclonal antibody that binds to activated factor IX (FIXa) and FX. It acts as a bridge between these two factors, mimicking the in vivo activity of FVIII, enabling the generation of activated FX (FXa), which is required to convert prothrombin to thrombin [29]. The HAVEN 1 and 2 trials led to emicizumab being approved for use in adults and children with severe HA and inhibitors (HAwI) in 2017 by the US Food and Drug Administration and in 2018 by the european medicines agency [30, 31]. These studies reported excellent safety and very low annualised bleeding rates (ABR) in patients above and below 12 years [32, 33]. The subsequent HAVEN 3 and 4 studies showed similar results in HA without inhibitors [34, 35], with regulatory approval granted shortly after [30, 31]. More recently, interim results from the HAVEN 6 study of the use of emicizumab in patients with mild and moderate HA (FVIII >5% and 1%–5%, respectively) have been reported, with 80% of patients remaining bleed‐free during the study period. Final results are awaited [36].

Emicizumab has a good safety profile. Anti‐drug antibodies (ADAs) with neutralising potential developed in two patients in HAVEN 2, of which one resolved without requiring treatment whilst no ADAs were observed in the other reported studies [33]. Three patients in HAVEN 1 developed thrombotic microangiopathy (TMA) which will be discussed later in this review. Two additional thromboembolic events were reported across 401 patients HAVEN 1–4, but no other participants discontinued following any other adverse events [37]. A further clinical dilemma yet to be answered is whether emicizumab can prevent the development of inhibitors in patients who have never received FVIII treatment. A summary of ongoing clinical trials of emicizumab and other novel therapies is shown in Table 1.

**TABLE 1:** Clinical trials of novel agents under investigation for persons with haemophilia (PWH)

Study	Phase	Information	Trial status
Emicizumab			
HAVEN 1 NCT02622321	3	109 HAwI patients ≥12 years randomised to weekly emicizumab (1.5 mg/kg subcutaneous) versus no prophylaxis	ABR 2.9 versus 23.3 (87% reduction in ABR)
HAVEN 2 NCT02795767	3	85 HAwI patients <12 years received weekly (1.5 mg/kg), alternate weekly (3 mg/kg) or monthly (6 mg/kg) emicizumab	ABR 0.3, 0.2, 2.2, respectively
HAVEN 3 NCT02847637	3	152 severe HA patients without inhibitors ≥12 years randomised to weekly (1.5 mg/kg) or alternate weekly (3 mg/kg) emicizumab, or to no prophylaxis	ABR 1.5, 1.3, 38.2, respectively (96%–97% reduction in ABR)
HAVEN 4 NCT03020160	3	48 severe HA with or without inhibitors, of all ages, received monthly emicizumab 6 mg/kg	ABR 2.4 for treated bleeds and 1.7 for treated joint bleeds
HAVEN 5 NCT03315455	3	Severe HA ≥12 years randomised to weekly emicizumab (1.5 mg/kg) or monthly (6 mg/kg)	Closed to recruitment
HAVEN 6 NCT04158648	3	20 mild and 51 moderate HA patients received weekly (1.5 mg/kg), alternate weekly (3 mg/kg) or monthly (6 mg/kg) emicizumab	Pre‐treatment ABR 2, post‐treatment ABR 0.8, 80% had no bleeds
HAVEN 7 NCT04431726	3	HA 0–12 months	Recruiting
NCT04205175	Single‐centre, open‐label	HAwI, evaluation of safe dose range of FEIBA	Closed to recruitment
NCT04303572	3	ITI with emicizumab versus ITI alone	Recruiting
NCT04188639	Multi‐centre, open‐label	Acquired HA	Recruiting
MIM8			
FRONTIER 1 NCT04204408	1/2	HA ≥12 years with or without inhibitors	Closed to recruitment
AT			
Fitusiran			
NCT02035605	1	17 patients with HAwI/HBwI received monthly fitusiran at 50 or 80 mg	Reduction of AT by 82%–87% ABR 0 versus 21.8, 65% of patients had no bleeds
ATALS‐A/‐B NCT03417245	3	93 HA and 37 HB patients ≥12 years randomised to 80 mg fitusiran versus no prophylaxis	ABR 0 versus 21.8 (90% reduction in ABR), 51% of the fitusiran arm had no bleeds with improved patient reported outcomes
ATLAS‐PPX NCT03549871	3	HA/HB ≥12 years	Closed to recruitment
TFPI			
Concizumab			
Explorer 3 NCT02490787	1	HA dose‐escalation study	Completed
Explorer 4 NCT03196284	2	9 HAwI and 8 HBwI patients received daily concizumab (0.15–0.25 mg/kg)	ABR 3 and 5.9
Explorer 5 NCT03196297	2	36 HA (no inhibitors) patients received daily concizumab (0.15–0.25 mg/kg)	ABR 7
Explorer 7 NCT04083781	3	HAwI/HBwI	Closed to recruitment
Explorer 8 NCT04082429	3	HA/HB	Closed to recruitment
Marstacimab			
NCT02974855	1	26 HA/HB patients with or without inhibitors, dose‐escalation study	ABR reduced by 85%–98% versus historical controls
NCT03363321	2	HA/HB with or without inhibitors, 150 or 300 mg weekly	ABR reduced by 84.5% versus 92.6%
NCT03938792	3	Severe HA, moderate–severe HB	Recruiting
APC			
SerpinPC			
NCT04073498	1/2	Healthy volunteers and severe HA/HB, safety and tolerability study	Closed to recruitment

Abbreviations: ABR, annualised bleeding rates; APC, activated protein C; AT, antithrombin; HA, haemophilia A; HB, haemophilia B; ITI, immune tolerance induction; TFPI, tissue factor pathway inhibitor.

There are three other FVIII mimetics under development. Mim8 (Novo Nordisk) is a next‐generation bispecific to FIXa and FX, which shows enhanced thrombin generation in vivo compared with sequence identical emicizumab analogue [38]. The FRONTIER 1 study is an ongoing phase 1/2 study of Mim8 in HA with or without inhibitors ≥12 years of age (NCT04204408). BS‐027125 (Bioverativ) is currently in preclinical evaluation with no human data yet available, as is NIBX‐2101 (Takeda, Japan).

## 4 NON‐FACTOR THERAPIES—REBALANCING AGENTS

Rebalancing therapies hinge on the concept that the bleeding phenotype of PWH may be alleviated by altering the balance of haemostasis in favour of a more pro‐coagulant state. Therapeutic agents in preclinical and clinical development currently fall into three categories: antithrombin (AT) inhibitors, tissue factor pathway inhibitor (TFPI) inhibitors and inhibitors of activated protein C (APC). One of the advantages of this type of non‐factor therapy over the mimetics is its effectiveness in both HA and HB with or without inhibitors.

### 4.1 Reducing antithrombin

AT is a glycoprotein that binds to and inhibits FXa and thrombin (FIIa). Fitusiran (ALN‐AT3, Sanofi) is a small molecule RNA interference (siRNA) agent, which acts by binding to and degrading the mRNA encoding AT. This leads to reduced AT levels and thus increased thrombin potential [39]. A phase 1 dose‐escalation study in 25 PWH (moderate–severe HA or HB) reported a dose‐dependent mean maximum AT reduction of 70%–89% from baseline, with a reduction of >75% enabling a similar degree of thrombin generation compared with healthy volunteers [40]. Initial results from the phase 3 ATLAS‐A/B study have recently been published, showing a 90% reduction in ABR in patients with severe HA or HB with or without inhibitors, who received fitusiran 80 mg once a month compared with daily factor prophylaxis [41]. Population pharmacokinetic and pharmacodynamic modelling data have since suggested that patients may be commenced on a lower dose of fitusiran, namely 50 mg every other month. Approximately 50% of patients will achieve sustained AT levels <35% at this lower dose, whereas the other 50% require dose escalation to 50 mg monthly. The authors suggest that only those with peak AT activity >35% receiving 50 mg monthly should be escalated to the 80 mg dose [42].

### 4.2 Tissue factor pathway inhibitor

TFPI is a serine protease inhibitor that regulates the initiation of thrombin generation by preventing the activation of FX by the TF‐FVIIa complex and blocking early forms of prothrombinase. Inhibition of TFPI is, therefore another means of enhancing thrombin generation. TFPI is comprised of three Kunitz‐type domains, each with a discrete function. K1 binds to TF‐FVIIa, K2 to FXa and K3 to protein S [43]. Approximately only 10%–30% of TFPI circulates freely in plasma, where it carries out its fibrinolytic activity [44].

Concizumab (Novo Nordisk) is a humanised IgG4 monoclonal antibody directed against the K2 domain. The phase 1b Explorer 3 study was a dose‐escalation study in 24 patients with severe HA without inhibitors. Concizumab appeared safe, and a dose‐dependent decrease in the level of unbound TFPI and an associated increase in thrombin generation was observed [45]. Health‐related quality of life measures, including physical function and pain scores, improved during the study period [46]. The phase 3 Explorer 7 and 8 studies were halted in March 2020 due to three non‐fatal thromboembolic events but later resumed in August 2021. Results are eagerly awaited.

PF‐06741086 (Marstacimab, Pfizer) is another anti‐K2 TFPI monoclonal antibody. A phase 3 study in severe HA and moderate–severe HB is recruiting (NCT03938792). Another anti‐K2 monoclonal antibody, MG113, showed restoration of thrombin generation in animal models [47] and is currently being investigated in a phase 1 study in healthy volunteers and PWH (NCT03855696).

The BAY1093884 (Befovacimab, Bayer) is a monoclonal antibody with specificity to the K1 and K2 domains of TFPI. A phase 1 study showed promising initial results [48] however, the phase 2 study was terminated after three patients developed cerebral thrombotic events, particularly as none of these patients had been receiving additional haemostatic therapy for a bleeding episode [49]. BAX499 (Takeda) was an aptamer which interfered with the K1 and K3 domains. Enhanced thrombin generation and reduced bleeding were shown in preclinical studies [50, 51]; however, the phase 1 study was terminated due to increased bleeding events. The cause of this was elucidated to be related to the release of intracellular stored TFPI into the plasma, alongside reduced clearance of active TFPI, leading to enhanced fibrinolytic activity [52].

### 4.3 SerpinPC

APC is an endogenous anticoagulant that acts by degrading FVa and FVIIIa. Patients with FV Leiden have resistance to APC‐mediated FVa degradation. The presence of this common mutation in PWH was noted to ameliorate bleeding severity over 20 years ago, providing early evidence of its potential as a target in haemophilia treatment [53, 54]. Naturally occurring APC inhibitors are members of the serpin (serine protease inhibitor) family, such as alpha1‐antitrypsin, plasminogen activator inhibitor 1 and protein C inhibitor [55, 56, 57]. Modification of these serpins was used to increase sensitivity and specificity. The resultant product, SerpinPC (ApcinteXLtd), promoted thrombin generation in vitro and improved haemostasis in a murine model of HB [58]. A phase 1/2 trial in healthy volunteers and PWH is ongoing (NCT04073498). A monoclonal antibody to APC (HAPC1573) has also recently shown promising results in a nonhuman primate study [59].

PS acts as a cofactor for APC in the inactivation of FVa and FVIIIa and of TPI in inhibiting FXa [60, 61]. One group has used monoclonal antibodies against PS and siRNA knockdown in a murine model, demonstrating increased thrombin generation and resistance to APC and TFPI [62]. Currently, no human data are available.

## 5 ARE THERE ANY CHALLENGES WITH NON‐FACTOR THERAPIES?

### 5.1 Factor VIII equivalence

As novel treatments are increasingly utilised in the management of PWH and VWD, several practical dilemmas are coming to the fore. Although these therapies vastly improve haemostasis in severe haemophilia, they do not normalise thrombin generation to the level of individuals without haemophilia. FVIII or FIX levels below the expected levels of 50% still cause increased bleeding with trauma or procedures. For this reason, PWH in the mild category still require factor raising therapies in these clinical situations. When it comes to the novel therapies, what would be useful to know is the parameter, ‘factor VIII (or IX) equivalence’ to determine how much additional factor raising treatments may be necessary before surgical procedures or in case of significant bleeding, and conversely whether minor procedures may proceed without any adjunct factor therapy. One group used thrombin generation tests to calculate the equivalent FVIII level achieved by 11 severe HA patients receiving emicizumab prophylaxis. All patients had an equivalent FVIII of >10%, with the majority achieving levels >20%. Of note, an inverse correlation between patient weight and equivalent FVIII level was observed [63]. Animal models have provided insight into FVIII equivalence for fitusiran and concizumab [64], suggesting an FVIII equivalence of around 20% in both cases, assuming AT is suppressed to approximately 30% of normal when considering fitusiran [65, 66]. Whilst a FVIII level of 20% will prevent the majority of spontaneous bleeds, these data indicate that the days of requiring additional factors or bypassing agents are not over. Furthermore, optimal management of bleeding and surgery in patients receiving these therapies requires consideration of additional factors, such as alteration in laboratory monitoring practices.

### 5.2 Thrombosis

One of the logical concerns of modulating the haemostatic pathway towards the procoagulant side is the risk of thrombosis. Three patients in HAVEN 1 developed a TMA. These patients had all been concomitantly treated with aPCC (FEIBA) at doses of >100 IU/kg/day to manage bleeding, whereas no episodes were reported in patients receiving rFVIIa during the study period. One proposed explanation is that FEIBA contains the targets of emicizumab, FIX/IXa and FX/Xa, and that excess substrate availability may have led to uncontrolled thrombin generation and subsequent thrombotic complications [67]. The use of FVIIa appears to be safer in this setting [68]. An ongoing clinical trial (NCT04205175) investigates the safe and effective dose range for aPCC in emicizumab‐treated patients with HAwI and bleeding.

Trials with fitusiran were temporarily paused in 2017 following a fatal cerebral sinus thrombosis in a HA patient in the phase 2 study who had received a high dose of rFVIIa to treat a bleed [69]. Revised guidelines were implemented with reduced doses of bypassing agents and factor concentrates recommended. No thrombotic events have been reported to date in phase 3 ATLAS‐A/B study [41]. Commencing patients on a lower fitusiran dose and only increasing if AT inhibition is insufficient may mitigate thrombosis risk [42].

The phase 3 Explorer 7 and 8 concizumab studies were halted in March 2020 due to three non‐fatal thromboembolic events. Results are awaited. However, the thrombotic signal seen in these studies is of concern, as these events were not associated with either aPCC or rFVIIa administration, both of which are known to independently increase thrombotic potential. Similarly, the production of BAY1093884 was halted entirely following the occurrence of three cerebral thrombotic events, also in patients who had not received bypassing agents or factor concentrates for bleeding [49]. Until more is known, these agents should be used with caution in patients with additional risk factors for thrombosis.

### 5.3 Laboratory monitoring

Novel therapies interfere with a number of standard coagulation assays [70]. The activated partial prothrombin time (APTT) is shortened, usually to within the normal reference range, even with subtherapeutic emicizumab levels, due to its effect on FIX and FX [71, 72]. Therefore, one‐stage FVIII assays are unsuitable, as are chromogenic assays that use human‐derived FIXa and FX. Assays using bovine FIXa and FX are required [70], however results may still be falsely elevated following factor administration, and laboratories are recommended to perform local verification of assays in use.

Fitusiran does not appear to affect APTT readings, suggesting that both one‐stage and chromogenic assays may be used to monitor factor VIII replacement [73]. However, the availability and efficacy of emicizumab for HA, and EHL FIX replacement in HB, mean that the patients most likely to benefit from fitusiran are the HBwI population, in whom bleeding is managed with rFVIIa. Determining whether a patient is sub‐ or supratherapeutically dosed with fitusiran currently requires an AT level, which does not assist clinicians when managing an acute bleed.

Thrombin generation and viscoelastic tests seem logical candidates for monitoring these novel agents [74]. Thromboelastography readings have been demonstrated to reflect improved haemostasis in a patient with severe HA commencing emicizumab [75]. Whether such tests might benefit predicting thrombotic outcomes is worth consideration. Emicizumab‐treated patients receiving high doses of aPCC are at risk of TMA [32], whereas thrombosis was seen following rFVIIa administration in a fitusiran study [69]. Thrombin generation and viscoelastic tests may be beneficial for patients requiring additional haemostatic therapy, in whom thrombin generation may rise unexpectedly.

### 5.4 Management of bleeding

**FIGURE 1:**
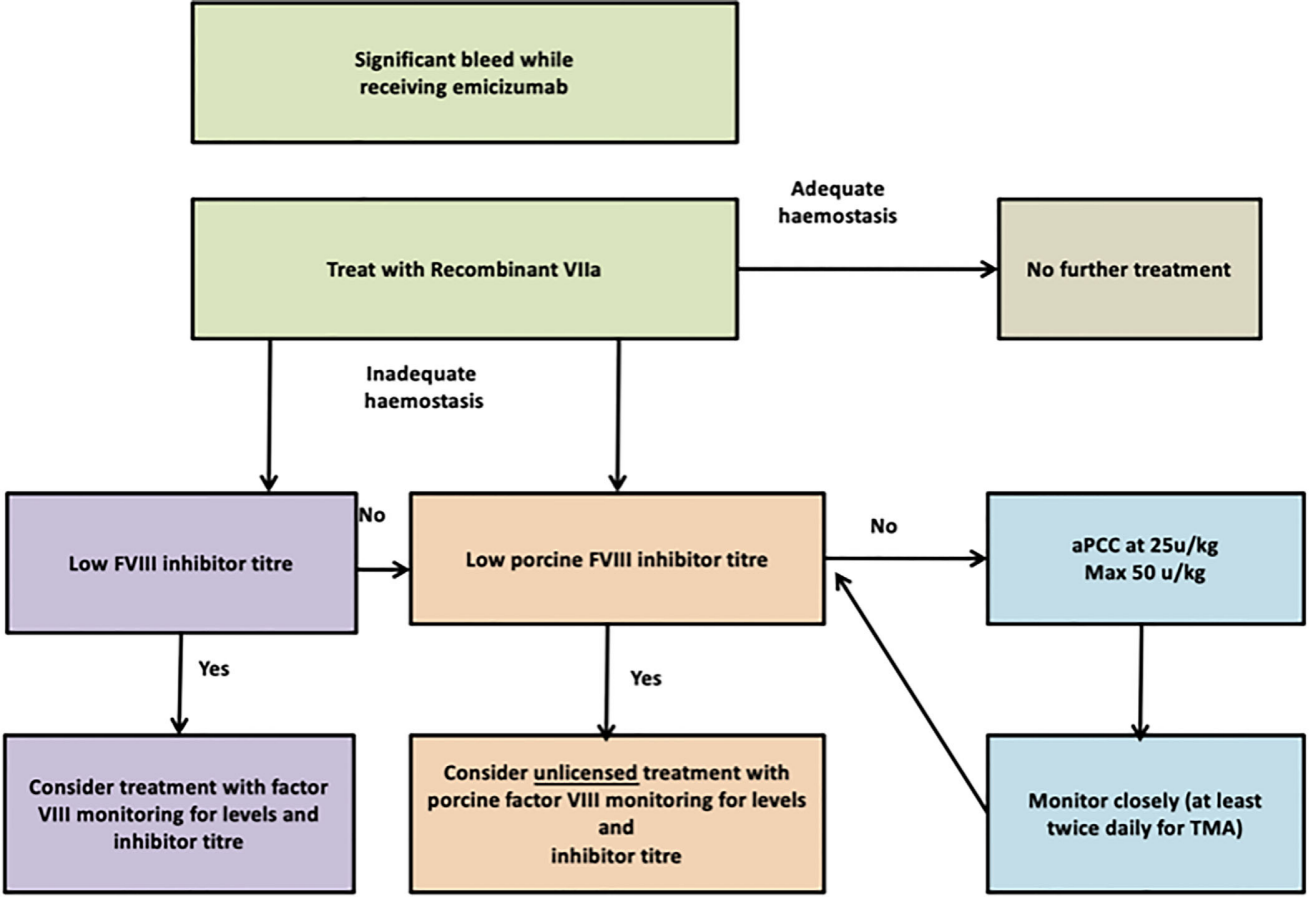
Management of bleeding in patients receiving emicizumab. Adapted from advice given by the UK Haemophilia Centre Doctors' Organisation guidelines for the management of bleeding in patients receiving Emicizumab

The UK Haemophilia Centre Doctors’ Organisation published guidance regarding the management of bleeds in patients with inhibitors receiving emicizumab in 2018, with a similar document issued by a German expert panel in 2021 [76, 77]. As patients have an FVIII equivalence of >10%, minor bleeds may resolve with tranexamic acid alone. For a major bleed, rFVIIa is the first‐line therapy, with an initial suggested dose of no higher than 90 μg/kg to avoid excess thrombin generation. If two‐hourly administration of rFVIIa at this dose does not secure haemostasis, options are FVIII concentrate in patients with a low inhibitor titre, off‐license use of porcine‐derived FVIII concentrate if available in patients with a low porcine inhibitor titre, or aPCC. Guidelines recommend a maximum single dose of 50 u/kg and a cumulative daily dose of 100 u/kg in such cases with close monitoring for TMA development. The HAVEN 3 study of emicizumab in non‐inhibitor patients did not give specific dosing guidance regarding on‐demand use of FVIII for bleeding. Comparison of management of bleeding events in 48 patients on HAVEN 3 versus 48 patients who received FVIII prophylaxis in a prior study showed fewer events required treatment, but the median dose of FVIII administered per treated bleed was similar (43.5 IU/kg in the FVIII prophylaxis group and 50.0 IU/kg in the emicizumab group) [78]. Current guidelines only consider inhibitor patients. Management of bleeding in patients receiving emicizumab is depicted in Figure 1. Future studies should consider looking at reduced doses of rFVIIa and FVIII in managing bleeding in patients with and without inhibitors receiving emicizumab.

The phase 2 fitusiran study protocol included dosing guidelines for bleed management, with reduced doses of FVIII, FIX, aPCC and rFVIIa specified (10 IU/kg, 20 IU/kg, 30 U/kg and ≤45 μg/kg, respectively). One hundred and seven bleeding events in 14 patients were assessed for compliance with these guidelines. A single reduced dose of factor or bypassing agent achieved satisfactory haemostasis in 60% of cases. There was substantial variation between patient subtypes, with one reduced dose proving sufficient in 92% of bleeds in HA without inhibitors, 78% in HAwI, 33% in HBwI and 25% in HB without inhibitors [79]. Currently, there is inadequate evidence available to guide management of bleeding episodes in patients receiving fitusiran or concizumab.

### 5.5 Perioperative management

Perioperative care of patients receiving novel therapies is another area of uncertainty. Retrospective analysis of HAVEN 1–4 identified 115 patients who underwent 215 minor surgeries, predominantly dental and central venous access device‐related procedures, and 18 patients who underwent 18 major surgeries, half of which were synovectomies or arthroplasties. Sixty‐six percent of the minor procedures were performed without prophylactic factor concentrate, of which 91% did not require treatment for bleeding. Bleeding was not seen more frequently in patients managed expectantly for bleeding than those who received preoperative factor. Fifteen of the 18 major surgeries were performed with prophylactic factor replacement, resulting in three bleeds, only one of which required treatment. Conversely, none of the three patients managed expectantly were reported to have bled [80, 81]. Considering fitusiran, a review of patients in phase 1 and 2 open‐label studies identified seven patients who underwent surgery [82, 83]. Most cases received prophylactic factor or bypassing agent with good outcomes reported however, limited data regarding dosing and duration of treatment required. The Explorer 4 and 5 studies of concizumab excluded patients requiring major surgery and did not permit systemic use of antifibrinolytics. A total of 50 procedures were carried out, of which 30 were dental. There were 15 surgery‐related bleeds, of which 14 were classified as mild or moderate. No information is provided regarding the management of these events. Of note, one patient in Explorer 4 had a total knee replacement without any bleeding [84].

**FIGURE 2:**
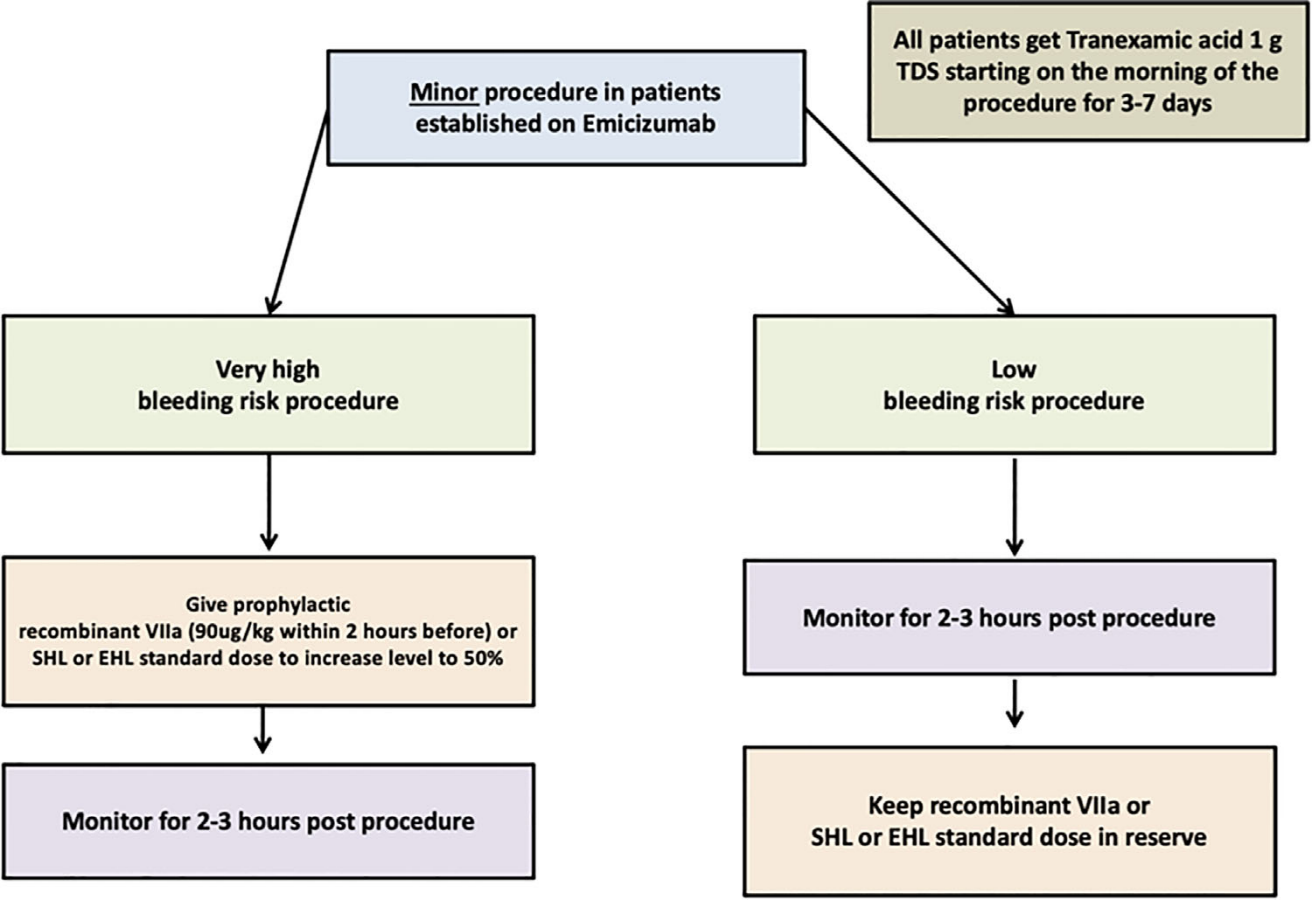
Management of minor procedures in patients receiving emicizumab

**FIGURE 3:**
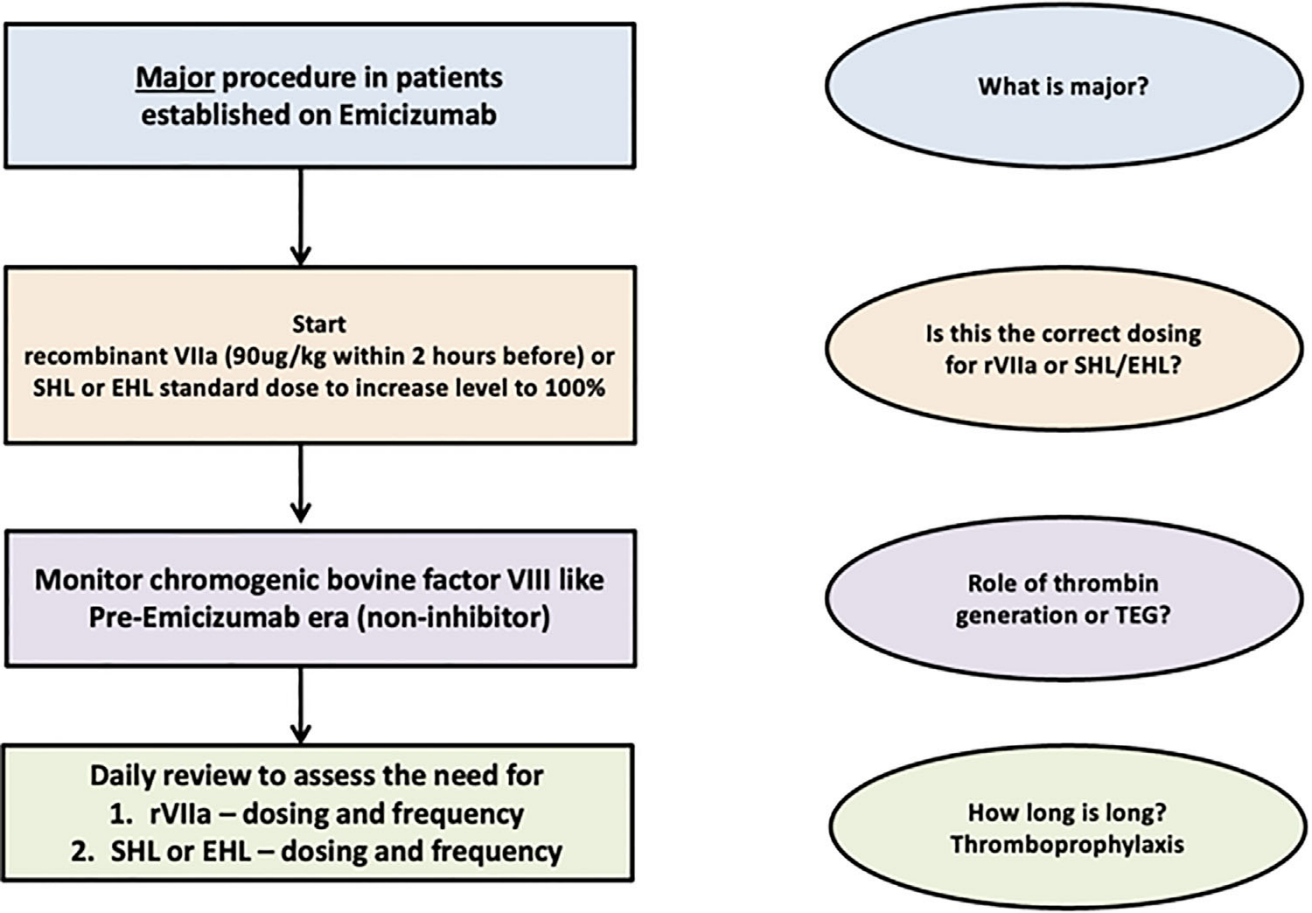
Management of major procedures in patients receiving emicizumab

In Figures 2 and 3, we provide a suggested treatment algorithm for patients receiving emicizumab who require surgery. For minor procedures with low bleeding risk, we recommend that rFVIIa (for patients with inhibitors) or FVIII concentrates (for those without inhibitors) may be reserved for use only in the event of abnormal bleeding. PWH undergoing minor procedures with a high bleeding risk should receive preoperative rFVIIa, or FVIII concentrate to achieve a bovine chromogenic FVIII level of 50%, and those undergoing major procedures should have a preoperative FVIII level of 100%. These recommendations are based upon available evidence, published guidelines and author opinion [85, 86]. Several important questions remain unanswered, such as how to best define major surgery in these patients, whether standard rFVIIa dosing is appropriate, how to safely use aPCC, whether viscoelastic tests may aid monitoring of perioperative haemostasis, how long supplemental FVIII or rFVIIa is required for and at what stage is thromboprophylaxis indicated.

Currently, there is inadequate evidence available to guide perioperative management of patients receiving fitusiran or concizumab. Further data are eagerly awaited.

## 6 VON WILLEBRAND DISEASE

### 6.1 Replacing factor VIII

Some of the bleeding tendency in patients with Type 3 VWD (<1% VWF activity) and Type 2N (Normandy) VWD (in which the mutation in VWF results in defective binding and stabilisation of FVIII) is accounted for by FVIII deficiency [87]. This cannot be solved by simply replacing FVIII, as the half‐life of exogenous FVIII is very short due to the patient's ineffective VWF. Equally, the use of FVIII/VWF concentrated in 2N patients may lead to excessively high VWF levels [88].

Emicizumab, as described above in PWH, has been used in a patient with Type 3 VWD, alloantibodies against both VWF and FVIII, and active bleeding [89]. A similar case series of two adults and two children with Type 3 VWD reported that prophylactic use of emicizumab reduced bleeding episodes and improved patient‐reported quality of life [90]. There are no formal studies of emicizumab in VWD, but this may warrant further investigation.

BIVV001 is a novel FVIII‐fusion protein being investigated in PWH. BIVV001 consists of a recombinant FVIII protein fused to the FVIII‐binding domain of VWF. This prevents endogenous VWF from binding whilst maintaining the VWF‐stabilising characteristics. The product also contains two XTEN polypeptides, which are unstructured hydrophilic polypeptides designed to further extend half‐life via steric shielding [91, 92]. A phase 1/2 study in HA reported a three to fourfold higher half‐life for BIVV001 than recombinant FVIII [93]. Another recent study in severe HA administered four weekly doses of BIVV001, with FVIII levels of 69% and 12% at 3 and 7 days after the final dose recorded [94]. BIVV001 could provide a new means of treating Type 2N VWD without leading to excess elevation of VWF levels.

### 6.2 Increasing VWF

**FIGURE 4:**
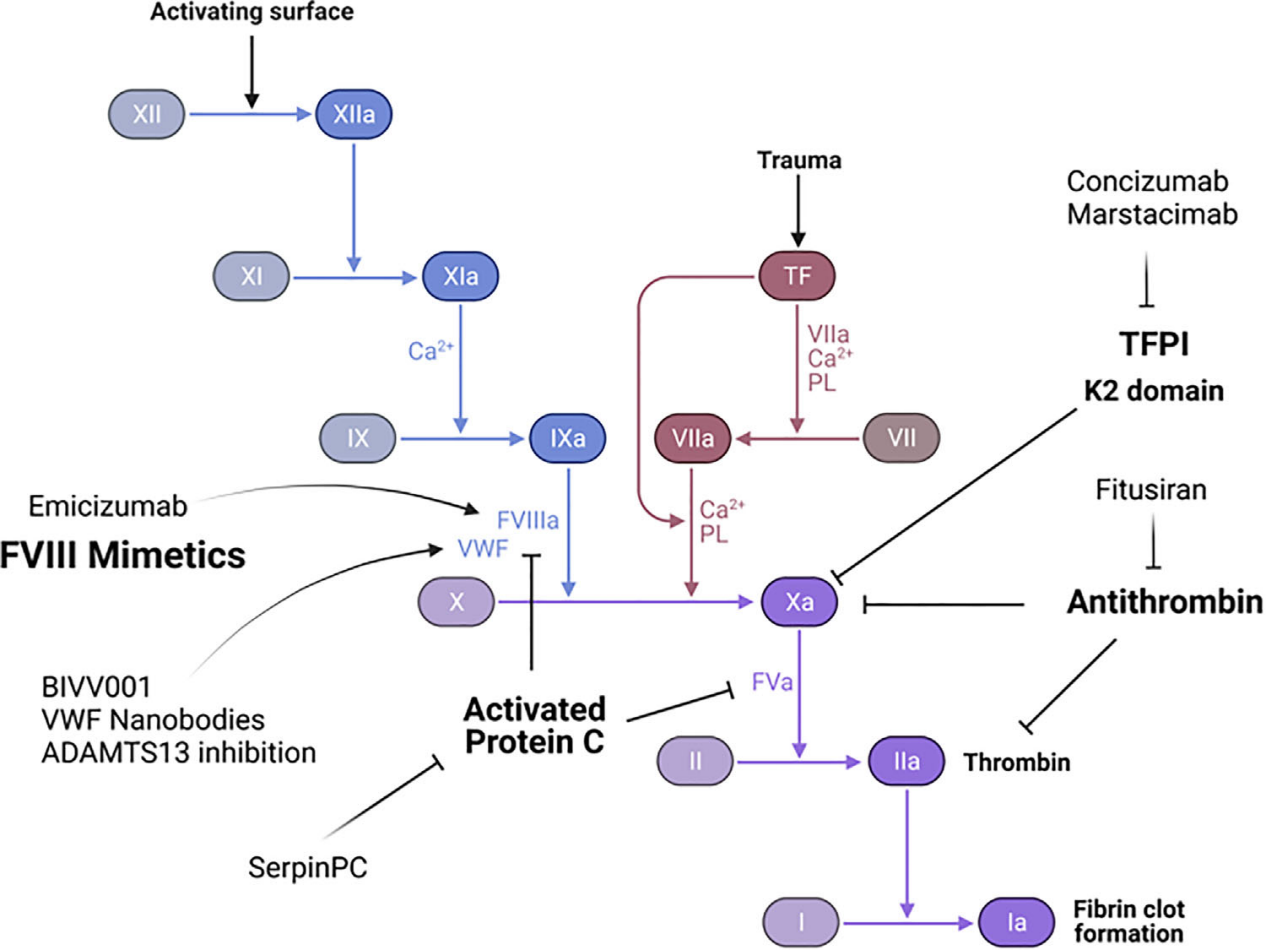
Novel treatments for persons with haemophilia (PWH) and von Willebrand disease (VWD)

A novel method of increasing endogenous VWF levels by prolonging half‐life is to use VWF‐specific nanobodies fused to an albumin‐binding peptide. These nanobodies bridge VWF to albumin, thus extending survival in the circulation. Using this technique, murine studies have shown a sevenfold increase in VWF half‐life. Another possible means of increasing VWF levels is to reduce the metalloproteinase ADAMTS13. The anti‐VWF monoclonal antibody Mab508 binds to the ADAMTS13‐docking site on VWF, preventing excessive degradation [95]. Further investigation is required to determine whether such therapies could prove efficacious in VWD without leading to complications akin to thrombotic thrombocytopenic purpura [96]. Figure 4 summarises the current pharmacological targets under development in PWH and VWD.

## 7 CONCLUSION

Management options for PWH have improved vastly over the last number of years following the development of EHL products and, more recently emicizumab. Other novel therapies are proving efficacious and generally safe in large‐scale clinical trials and are likely to be available for general use over the following number of years. Progress in VWD has been less dramatic, with patients still generally relying upon VWF concentrates. Although these novel therapies demonstrate significant reductions in ABR compared with standard prophylaxis, bleeding events still occur, and optimal management in these cases can be complicated by the presence of the novel therapy. Causes of potentially catastrophic complications, such as TMA associated with emicizumab and aPCC, are not fully known and require further study1. Moreover, as treatment continues to improve, how efficacy is measured in clinical trials needs to develop alongside pharmacological advances. Patient‐reported outcomes and quality of life measures are of paramount importance in future studies in order to improve further outcomes for PWH and other bleeding disorders [97].

## CONFLICTS OF INTEREST

Dawn Swan had no conflicts of interest.

## FUNDING INFORMATION

Johnny Mahlangu—Honoraria, consulting fees, speakers’ bureau fees and research funding: CSL Behring, Catalyst Biosciences, Freeline Therapeutics, Novo Nordisk, F. Hoffmann‐La Roche Ltd., Sanofi, Spark Therapeutics Inc., Takeda. Jecko Thachil—Honoraria: Roche Chugain, Octapharma, Sobi, Takeda, Novo Nordisk.

## AUTHOR CONTRIBUTIONS

Dawn Swan wrote the manuscript and provided Figure 4. Johnny Mahlangu critically appraised the manuscript. Jecko Thachil conceived the review, critically appraised the manuscript and provided Figures 1, 2, 3.
